# Agenda-Setting for COVID-19: A Study of Large-Scale Economic News Coverage Using Natural Language Processing

**DOI:** 10.1007/s41060-022-00364-7

**Published:** 2022-10-06

**Authors:** Guang Lu, Martin Businger, Christian Dollfus, Thomas Wozniak, Matthes Fleck, Timo Heroth, Irina Lock, Janna Lipenkova

**Affiliations:** 1 Institute of Communication and Marketing, Lucerne University of Applied Sciences and Arts, Zentralstrasse 9, Lucerne, 6002 Switzerland; 2 Institute of Language Competence, ZHAW Zurich University of Applied Sciences, Theaterstrasse 17, Winterthur, 8401 Switzerland; 3 Institute of Financial Services Zug, Lucerne University of Applied Sciences and Arts, Zentralstrasse 9, Lucerne, 6002 Switzerland; 4 Institute of Communication Science, Friedrich Schiller University Jena, Ernst-Abbe-Platz 8, Jena, 07743 Germany; 5 Anacode GmbH, Kurfürstendamm 76, Berlin, 10709 Germany

**Keywords:** COVID-19, News media coverage, Business and society, Economic impact, Big data analysis, Natural language processing (NLP)

## Abstract

Over the past two years, organizations and businesses have been forced to constantly adapt and develop effective responses to the challenges of the COVID-19 pandemic. The acuteness, global scale and intense dynamism of the situation make online news and information even more important for making informed management and policy decisions. This paper focuses on the economic impact of the COVID-19 pandemic, using natural language processing (NLP) techniques to examine the news media as the main source of information and agenda-setters of public discourse over an eight-month period. The aim of this study is to understand which economic topics news media focused on alongside the dominant health coverage, which topics did not surface, and how these topics influenced each other and evolved over time and space. To this end, we used an extensive open-source dataset of over 350,000 media articles on non-medical aspects of COVID-19 retrieved from over 60 top-tier business blogs and news sites. We referred to the World Economic Forum’s Strategic Intelligence taxonomy to categorize the articles into a variety of topics. In doing so, we found that in the early days of COVID-19, the news media focused predominantly on reporting new cases, which tended to overshadow other topics, such as the economic impact of the virus. Different independent news sources reported on the same topics, showing a herd behavior of the news media during this global health crisis. However, a temporal analysis of news distribution in relation to its geographic focus showed that the rise in COVID-19 cases was associated with an increase in media coverage of relevant socio-economic topics. This research helps prepare for the prevention of social and economic crises when decision-makers closely monitor news coverage of viruses and related topics in other parts of the world. Thus, monitoring the news landscape on a global scale can support decision-making in social and economic crises. Our analyses point to ways in which this monitoring and issues management can be improved to remain alert to social dynamics and market changes.

## Introduction

When the first news reports on COVID-19 were published in the early 2020s, one could not have predicted how profoundly the virus would impact the global economy and shape our daily lives. Beginning primarily as a health issue, COVID-19 has become a “major player” in business and society [1]. This shift is reflected not only in economic figures, but also in the plethora of news articles published around COVID-19. This language data is the starting point of our study.

The pandemic “has substantially increased news consumption for mainstream media” as is shown in a survey conducted in 2020 in the U.K., the U.S., Germany, Spain, South Korea and Argentina [2]. People search more intensively for information to make sense of a new, disruptive situation like a crisis [3]. As agenda-setters, journalists fuel and respond to these information needs [4]. But in the case of COVID-19, news media’s herd behavior cascaded in overreporting on the topic, such that the longer the pandemic lasted, the more people became “coronavirus-news-fatigued” [3, 5]. Decision-makers in organizations engage in issue management to cope with exogenous challenges such as those posed by the pandemic crisis. To this end, policymakers or managers in large corporations follow the news media to gather information and prepare for potential problems [6]. While COVID-19 is, at its heart, a medical subject, it has induced several facets stretching from its impact on the economy to business management to even arts and culture [7–9]. Particularly with the outbreak of the pandemic, businesses and policymakers were concerned about potential economic and resulting social impacts such as layoffs, bankruptcies, and economic downturns. Therefore, it is worthwhile analyzing the specifics of economic news on COVID-19, how they have changed over time and across the world in response to COVID-19 cases along the following research questions (RQs):

**RQ1** Which economy-related topics were most prevalent in the news between January and August 2020, and which did *not* surface and *why*?

**RQ2** How have these topics and their corresponding subtopics developed and influenced each other *over time* and *around the world* parallel to COVID-19 cases?

We explored these questions on the basis of an open-source dataset provided by Anacode^1^ consisting of more than 350,000 media articles related to COVID-19, spanning the entire year 2020. Focusing on the non-medical aspects of COVID-19, these articles include a wealth of economic information and allow to extract valuable insights for organizations and companies. They were analyzed with a novel and comprehensive text analysis pipeline using natural language processing (NLP) techniques. The NLP techniques we used are not limited to topic modeling but extend to a wide range of advanced methods incorporating state-of-the-art sentence embedding models. The current pandemic is certainly not the last that humanity will experience, but rather one of several similar outbreaks that we will see [10]. Our contribution is to show in an exemplary way how to make strategic use of language data. Our method can also be applied in future crises, both by scientists, as it is based on advanced, modern NLP techniques, and by companies or policymakers.

A recent review of COVID-19 modeling found that studies on the economic impacts of COVID-19 are still very preliminary and lack a comprehensive analysis of economic-related variables, activities and issues [11]. Our work contributes to filling this research gap and is innovative in three ways. First, to the best of our knowledge, our paper is the first study to systematically explore a very large set of news articles belonging to different media outlets from an economic perspective, both temporally and spatially. Second, we leverage the World Economic Forum’s (WEF) Strategic Intelligence taxonomy to categorize media articles into different topics. Through a temporal analysis of the dissemination of news items in relation to their geographic focus, we find interactions between the identified socio-economic topics and link them to the cases of coronavirus reported by Our World of Data^2^. Third, we clarify the importance of global monitoring of the news landscape and management of a range of social and economic dimensions that may help prevent a social and economic crisis similar to COVID-19. We discuss the herd behavior of news media when independent news outlets report on the same topics during a global public health crisis. We note that in a pandemic such as COVID-19, more pluralistic press environments allow for more diverse viewpoints to be inserted in democratic discussion on societal issues.

We proceed as follows. In Section 2, we introduce selected related work. Section 3 describes the dataset and our text analysis pipeline. The NLP techniques we adopted are outlined in Section 4. Section 5 reports the results. Afterwards, we discuss the contributions and limitations of our findings and approaches in Section 6. The final Section 7 provides a conclusion and a brief outlook for future research.

## Related Work

The enormous social and economic consequences of COVID-19 have particularly attracted many researchers outside the medical field to study the wealth of information available online. The aim is to provide immediate reflection and a better understanding of the different aspects of the pandemic and its impact [12, 13]. Social media and news articles are excellent sources for this type of research, and some datasets are even open access [14–16]. The study of these materials has strongly contributed to the identification of fake news [17–28], thus providing a basis for management decisions by government and business.

In this case, a critical researcher may wish to read a variety of texts to get a comprehensive view of COVID-19, which is obviously a time-consuming task, despite the convenience of recommendation algorithms on news sites and the like [29]. The need for a varied diet of content would be better met if researchers had quick access to condensed information compiled by a more objective approach. Therefore, NLP techniques have been widely used to mine COVID-19 related text data [30–35] belonging to different types, such as scientific literature [12, 36–38], social media posts from e.g., Sina Weibo [39–41], Reddit [42–44] and Twitter [40, 45–58], and news articles [59–62]. Many language comprehension tasks can be effectively solved with NLP, including information retrieval, misinformation detection, literature-based discovery, question answering, topic modeling and sentiment analysis [30, 63–66].

In the literature, a large body of research has focused on using Twitter data to understand public discourse about COVID-19 because of its accessibility. The main NLP techniques used, among others, include neural and non-neural topic modeling [67]. Typical existing studies aim to extract useful clinical and health-related information in Twitter data using topic clustering and sentiment analysis [50, 53, 56, 68]. Some efforts have been spent on monitoring the different topics generated from Twitter and their development over time [49, 51, 55, 58]. Here, a remarkable work was done by Ordun et al. [51], who used topic modeling and clustering techniques to extract 20 different topics related to health and disease. Importantly, the retweet time was calculated to understand how quickly the COVID-19 message spread on social media. It is worth noting that the above research on Twitter data is not only at the individual level, but also extends to government agencies [69, 70] and corporations [71] to obtain their responses to COVID-19. These official communications are essential for crisis management.

In contrast to social media, which is often conflated with fake news, the official news media is considered a professional and highly credible source of information but remains surprisingly under-researched in the context of COVID-19 [11]. Some studies have attempted to extract the specific information in the news to predict the transmission of the virus [72] and the results of the COVID-19 diagnostic test [73]. Most of the existing relevant studies utilized similar topic modeling methods to detect topics as well as sentiments and compared coverage between news media and social media [74, 75]. The topics extracted were usually described as static and health-related, with very few exceptions. For example, Ghasiya and Okamura [76] sought to identify the most prevalent topics in newspapers from four countries, i.e., the U.K., India, Japan and South Korea by analyzing a database consisting of over 100,000 COVID-19 news headlines and articles using the topic modeling approach Top2Vec. The results showed, at a preliminary level, several hot topics of the time, such as education, economy, the U.S. and sports. Leveraging automated text summarization and article clustering, Wan et al. [77] collected 48,765 articles from 10 U.S. media outlets for the time span of 1 January 2020 to 4 April 2020 and modeled the development of topics over time. The framework by Wan et al. [77] consisted of three building blocks: extracting text summaries, clustering the summaries, and identifying topics on the basis of clustering. Although a large number of articles have been substantially refined using the summarization technique, some general information in the articles that affects the clustering results may have been lost. Agade and Balpande [78] used the same dataset as ours, but for a shorter period, from January 2020 to May 2020, to explore the non-medical aspects of COVID-19 as reported in the news. This study was limited to static topics and lacked a discussion of the economic implications of the findings. More recently, Dörr et al. [79] developed a sophisticated three-stage framework, i.e., initial web analysis, subsequent business surveys and retrospective analyses of company activities, with the aim of helping decision-makers find empirically informed responses to economic crises caused by the COVID-19 pandemic. In the NLP-based part of their framework, however, the authors limited themselves to news published by corporate websites, and thus to self-reported information by the companies themselves. Furthermore, it is noticeable that the NLP techniques used in the research of Dörr et al. [79] and similar ones [26, 27, 44] are mainly based on pretrained language models that may or may not be supported by a fine-tuning step. This seems to be the state of the art in COVID-19 related modeling of news data.

News media are agenda-setters in public discourse because they pre-select topics and multiply them through their publication [4]. News topics usually follow a wave pattern that corresponds to real events, such as COVID-19 cases, and are triggered by the adaptive behavior of journalists [80]. Given the increasing economic pressures on the media industry and the limited carrying capacity of the media system [81], journalists are incentivized to respond to readers’ information needs rather than to develop independent agendas. Editorial decisions in online news are driven by news values such as the short-term relevance of a story or whether it links to news stories from the past, the latter reinforcing the publication of stories on similar news topics within and across media [82]. In consequence, researchers and consumers have observed herd behavior and cascading effects, where news media tend to imitate each other and report on the same topics that promise clicks or sales [83]. However, this leads to a less heterogeneous news environment where less click-heavy topics get marginalized. These issues are rarely investigated in the context of COVID-19.

## Dataset and Text Analysis Pipeline

This section presents our dataset and explains how we analyzed it to achieve the research objectives. All the analysis was done in Python.

**Table 1:** Metadata in our dataset, illustrated with sample articles

Author	Date	Domain	Title	URL	Content	Topic area
Thomas Hughes	2020-01-02	Marketbeat	Three Industrial Giants You Should Own In 2020	https://www.marketbeat.com/originals/three-industrial-giants-you-should-own-in-2020/	With the end of the year just around the corner, it’s past time to think about positioning for 2020...	Business
Steve Anderson	2020-01-03	Marketbeat	Tesla (TSLA) Breaks Shipment Record, Beats Estimates for Fourth Quarter Vehicles Shipped	https://www.marketbeat.com/originals/teal-breaks-shipment-record-best-estimates-for-fourth-quarter-vehicles-shipped/	It could be forgiven, that some might think that Tesla (NASDAQ: TSLA) was little more than a big pile of pie-in-the-sky nonsense...	Business
Roberto Torres	2020-01-03	Ciodive	On the road to AI adoption, execs grapple with expertise and data	https://www.ciodive.com/news/ai-adoption-execs-expertise-data/569747/	CIOs kicked off 2019 with AI as an item to watch in the competition agenda...	Tech
Alden Wicker	2020-01-06	Instyle	Red Carpet Sustainability After Coronavirus Should Be Top Priority	https://www.instyle.com/fashion/red-carpet-coronavirus-sustainability-conversation	When the coronavirus pandemic is over and life returns to normal, celebrities will again walk red carpets for premieres and parties and awards shows...	Consumer

**Fig. 1:**
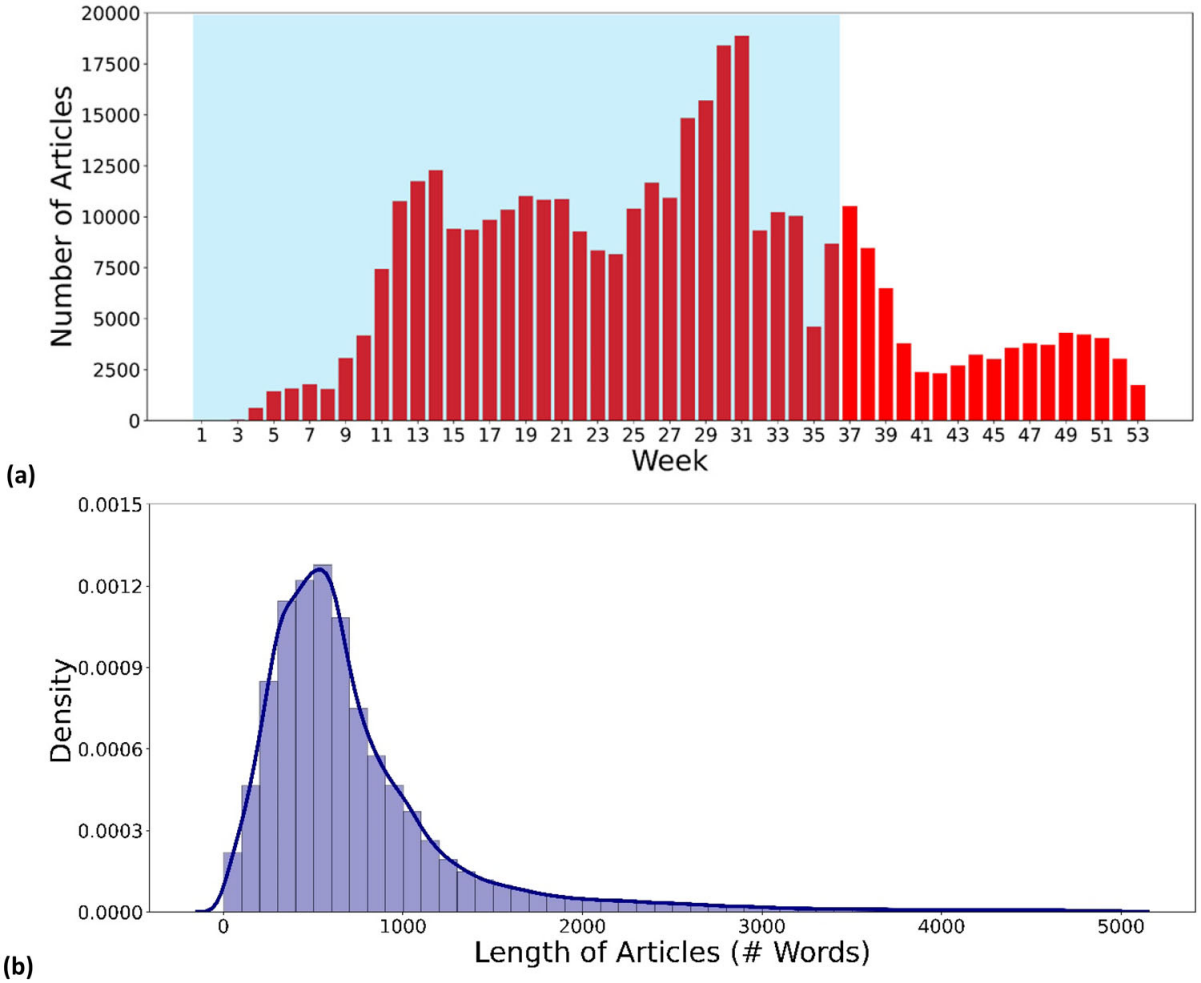
Media attention for COVID-19. (**a**) Number of articles in the collection viewed by different weeks in 2020. Articles published up to the end of August 2020 (highlighted in blue) are the focus of this study. (**b**) Density distribution of all article lengths. For visualization purposes, a cut-off is made at a length of 5,000 words

### Characteristics of the Dataset

The dataset used in our study is the open-source “COVID-19 Public Media Dataset by Anacode”, which comprises approximately 2-GB, in total 369,047 media articles scraped from 66 high-impact English blogs and news websites in 2020. This study focuses on data collected between January and August 2020, covering the first eight months of the pandemic, including the initial lockdown, the summer release period and the start of the second wave of the virus worldwide, i.e., a full pandemic cycle. The main data type is in text form, which is intended to represent the public media voices on COVID-19 during this period. Unlike other open-source datasets such as the “COVID-19 Open Research Dataset Challenge”^3^, our dataset focuses on the non-medical aspects of COVID-19 and its impact on society. Table 1 illustrates the metadata available for each article, including author, publication date and domain, title, URL, content and topic area. In this study, we focused on the analysis of the information “title” and “content”.

To get an overview of how many articles were collected each week in 2020, Fig. 1a plots the number of articles in the collection as a function of week. The first week runs from 1 January 2020 (Wednesday) to 5 January 2020 (Sunday). Relatively few COVID-19-relevant articles were published at the beginning of 2020, as COVID-19 took place mainly in China. From week 9, i.e., the end of February, there was a clear signal that public attention was being drawn to COVID-19 as the disease began to spread to other parts of the world. This trend continues until week 31, i.e., the beginning of August. After that, the number of articles collected decreases. Fig. 1b shows the density distribution of article length, quantified in words. It can be seen that most articles are less than 1,000 words long. For visualization purposes, the article length of 5,000 words was used as the cut-off point, which corresponds to about 98.4% of all articles in the original dataset.

**Fig. 2:**
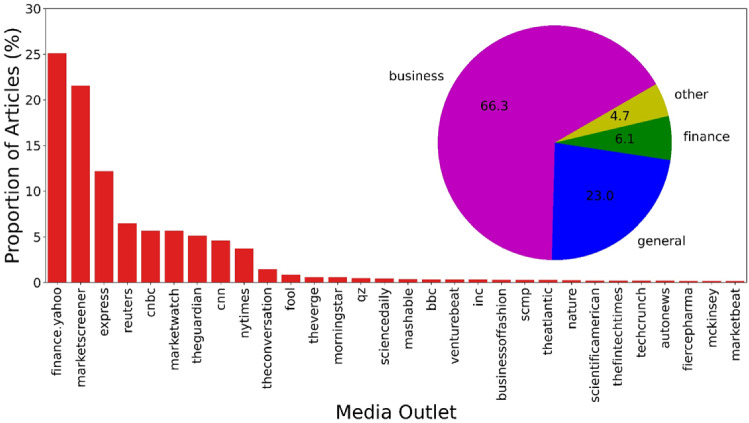
Proportion of articles per publication source. For visualization purposes, a cut-off point is set at the publication source MarketBeat to avoid a long tail. The inset shows the proportion of articles in each topic area as defined by Anacode. There are 11 topic areas, with the subordinate topics, i.e., *tech*, *science*, *consumer*, *healthcare*, *automotive*, *environment*, *construction*, and *artificial intelligence (AI)*, grouped under “other”

Fig. 2 shows the proportion of articles per publication source. There is considerable variation in the distribution across sources, with the most articles coming from Yahoo Finance (92,605), MarketScreener (79,551), express.co.uk (44,942), Reuters (23,861), CNBC (20,968), MarketWatch (20,962), TheGuardian (18,953), CNN (17,006), The New York Times (13,784), The Conversation (5,352) and The Motley Fool (3,154). The remaining sources have a relatively low distribution of articles (< 2,500 each). It should be noted that most of these sources, as the names of their media outlets imply, cover a broad range of topics. The distribution of article topic areas defined by Anacode is shown by the inset in Fig. 2. It can be seen that *business*, *general* and *finance* together account for over 95% of the articles.

### Preparing and Preprocessing of the Dataset

We took the following three steps to prepare and preprocess the dataset so that the text meets the input requirements of our analysis pipeline.

First, we analyzed the 290,000 articles published up to the end of August 2020. A quick check noted that about 1.4% of these articles were duplicates. The reason for these duplicates is that the articles were reproduced on different media platforms. This shows that the content of these articles is very much related to current events. We have therefore decided not to remove them from our dataset.

Second, we preprocessed the “title” and “content” of the article using Python and the Natural Language Toolkit (NLTK) [84]. This means that irrelevant site names, numbers, punctuation, stop words, line indicators and words with less than 3 letters were removed. We then lemmatized the remaining words, which helped us to identify important words from a clean, meaningful text.

**Fig. 3:**
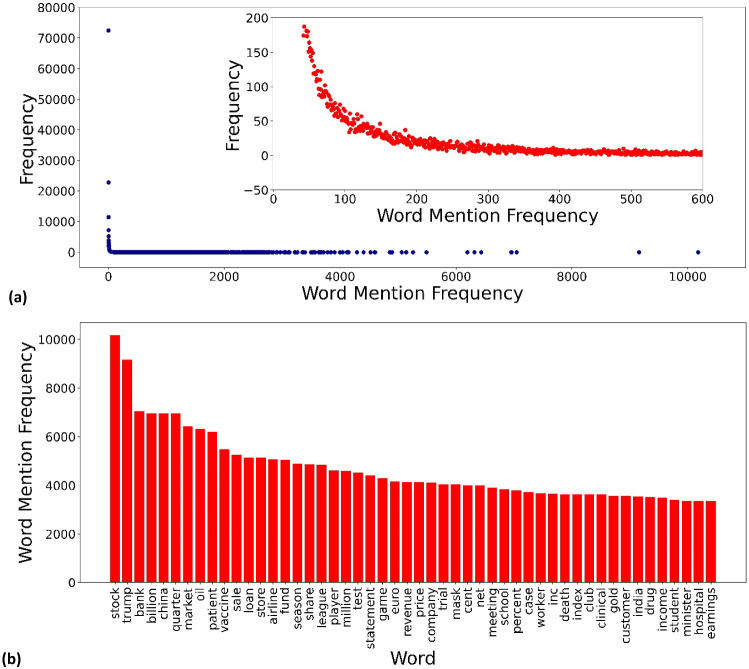
Extraction of the most important words from our dataset. (**a**) Frequency distribution of the word mention frequency. The inset shows a strong transition in the frequency of word mention around 100. (**b**) Frequency of mention of the 50 most important words. These words clearly indicate the underlying social and economic topics in our dataset

Third, we used the term frequency - inverse document frequency (TF-IDF) algorithm implemented in the Python module TextBlob [85] to extract the characteristic words in the dataset. From these characteristic words we can identify the main topics of the articles. However, applying the TF-IDF algorithm directly to our dataset of 290,000 articles is almost impossible as it takes an unacceptably long time to compute. Therefore, we divided the dataset into contiguous, smaller blocks, i.e., each block contained 500 articles, giving us a total of 580 blocks. We performed a TF-IDF analysis for each individual article block to identify the characteristic words belonging to each block and combined the results to filter out the most important words. With this procedure, the evaluation of the importance of words in many documents is possible and the calculation time was acceptable. To determine the characteristic words in each block, only the words with a TF-IDF score above the quantile of 0.95 were considered. To identify the most important words within the whole dataset, we calculated the frequency distribution of the word mention frequency based on the TF-IDF results. As can be seen in Fig. 3a , there is a sharp transition in word mention frequency around 100. We used 300 as the cutoff point and kept only the most frequently mentioned words. This reduced the original 162,127 characteristic words found to 2,669 of the most important words. Fig. 3b shows the first 50 such words. Most of these words are closely related to social and economic topics, which fit well with the content of our dataset and demonstrate the effectiveness of the TF-IDF algorithm used.

### Text Analysis Pipeline

**Fig. 4:**
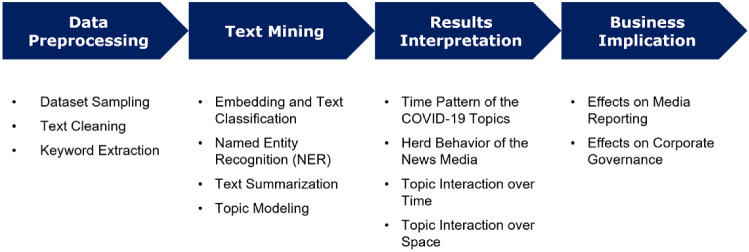
Text analysis pipeline proposed in this study. The text mining part is mainly based on transformer-based language models. The entire analysis code can be found on GitHub: https://github.com/lugulugu/COVID-19-News-Articles

To solve the text analysis task, we proposed the following investigations. On the one hand, we identify the main topics of the dataset from an economic perspective and show their dynamics in time and space. On the other hand, we explore potential links between these topics and relate them to epidemiological developments since 2020. The following NLP techniques were used: transformer-based sentence embedding, named entity recognition (NER), text summarization and topic modeling.

Fig. 4 shows the text analysis pipeline. We started by grouping each article into predefined topic groups using transformer-based sentence embedding so that we knew how many articles were collected under each topic for a given week in 2020. Subsequently, NER techniques were used to determine the spatial distribution of these topics over a given time window. Finally, the articles on specific topics were summarized, and we applied topic modeling to the grouped article summaries to identify micro-topics. In the course of our research, we tried to link the identified topics and topic relevance to extra-textual COVID-19 related events and derive useful implications for business and society.

### COVID-19 Topics Defined by the World Economic Forum

The World Economic Forum (WEF) website is an important place to gather COVID-19 related media releases. The WEF has developed COVID-19 topic networks^4^ to understand the complex forces during the pandemic. We have used the definitions and descriptions of the scope of these topics because they bundle a variety of socio-economic aspects of the pandemic into a manageable number of topic categories. To this end, the topic *COVID-19* is addressed through nine different issues: *COVID-19 Treatments*, *New Variants*, *The Media’s Role During COVID-19*, *Public Policy, Governance and COVID-19*, *COVID-19’s Economic Impact*, *The Virus and the Disease*, *COVID-19, Seasonality and Weather*, *Developing and Distributing Vaccines*, and *COVID-19’s Impact on Travel and Trade*. These key issues build what we call the Topic Level I. Each key issue is further linked to several topics at a finer level, i.e., the Topic Level II, which comprises in total 45 different topics ranging from governance and economics to arts and culture. Note that the links between the Topic Level I and II are not exclusive, i.e., a topic in the Topic Level II may be relevant to several topics in the Topic Level I. Therefore, the topics in the Topic Level II are linked according to their relevance. Interested readers can visit the *COVID-19* topics grouped in the global issues on the WEF website to see the two levels of topics and detailed text descriptions explaining the scope of each topic. As the Topic Level II covers very broad subjects, we have selected only those topics that are closely related to economic aspects.

## NLP Analysis Techniques

In this section we will briefly introduce the NLP techniques and our model used in the text analysis pipeline.

**Fig. 5:**
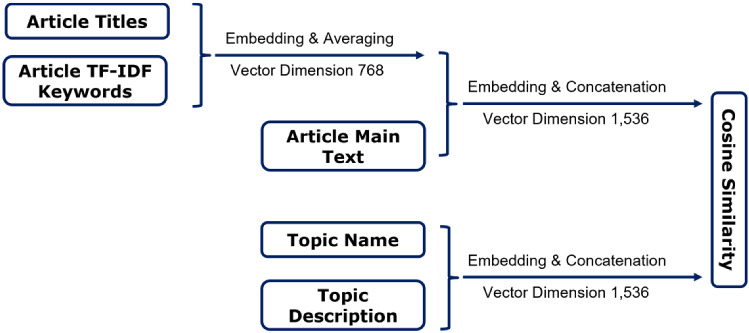
Application of the Sentence-BERT embedding to the articles and the topics. The average embedding vector from the article title and TF-IDF keywords is concatenated with the embedding vector for the main text of the article. In addition, topic names and descriptions are embedded and concatenated. The cosine similarity between the two final embedding vectors indicates the topic of the article under investigation

### Transformer-based Sentence Embedding - Sentence-BERT

The transformer-based sentence embedding model sentence-BERT [86] has been used to encode texts in a continuous, high-dimensional vector space due to its high accuracy and speed. Based on the cosine similarity of these vectors, texts can then be classified into different groups based on their semantic similarity. On the one hand, for each article, we embedded the title and the most important words contained in that article. The two vectors of dimension 768 obtained were averaged. Moreover, this new vector was concatenated with the article embedding vector of the same dimension, resulting in a final vector of dimension 1,536. In this way, we aim to capture the key messages of the titles and the most important words, as well as the general contextual information of the whole text. On the other hand, for each predefined topic, we concatenated the embedded topic name with the embedded text description of the topic from the WEF website, resulting in a final vector of dimension 1,536. By calculating the cosine similarity of the two final vectors, we were able to determine whether and to what extent the articles studied reported on the target topic. Fig. 5 illustrates the sentence embedding process.

Sentence-BERT is a modification of the BERT network [87], which inherits the constraint of the network on the length of the input text. To address this problem, we adopted a “divide-and-conquer” approach by dividing long articles and topic descriptions into sections of 100 words each. We embedded each section of text and averaged these vectors to obtain the final embedding of the corresponding long text. In this study, we used the pretrained sentence transformer model “distilbert-base-nli-mean-tokens” from the Hugging Face library^5^ [88]. To validate the obtained text embeddings, we also used the LASER embedding from Facebook Research [89] pretrained with long short-term memory (LSTM) networks that have no restriction on the length of the input text. Text classification results show that topic weights vary similarly over time between Sentence-BERT embedding and LASER embedding. Since Sentence-BERT contains a more powerful transformer model and runs faster, we used this technique to encode the text for further analysis.

### Named Entity Recognition (NER) - spaCy

We applied spaCy [90] NER techniques to the articles to identify potential geographic locations and organizations. The spaCy NER is more advantageous compared to many deep learning-based NER models [91] because it is rooted in industrial applications and easy to implement. We plotted the frequency of mentions of these locations and organizations as dots on a world map using the Python module GeoPandas [92], so that we can clearly see the hotspots associated with COVID-19 in 2020 and their changes over time. We counted how many times a particular geographical location or organization was mentioned in the text and scaled the size of the dot according to the frequency of its mention in the corresponding geographic coordinates.

### Text Summarization - Bert Extractive Summarizer

Text summarization is a conditional text generation technique [93] that can be further differentiated into extractive and abstractive summarization [94]. In this study, we used the Bert Extractive Summarizer [95], which uses the pretrained BERT model [87] to embed sentences in documents. This method clusters all sentences in a document to identify those closest to the center of the clusters, i.e., the most representative sentences. Due to the limitation of the model to the length of the input text, we have truncated the article to the first 1,000,000 characters. The length of the summary can be controlled by the minimum and maximum length of the output in words, which is set to 100 and 200 characters respectively. The resulting summary is expanded by concatenating it with the corresponding article title to ensure that the main content is preserved. Finally, we prepared a list of 290,000 summaries for our dataset.

### Topic Modeling - Top2Vec

The technique of topic modeling was used to identify further potential micro-topics from the grouped articles and relate them to actual events. Instead of using traditional generative probabilistic models such as Latent Dirichlet Allocation (LDA), which require assumptions about the number of topics in an article and an adjusted list of stop words, stemming and lemmatization [96], we used a more recent advance in the field, Top2Vec, which overcomes the above drawbacks of LDA and allows for a more semantically meaningful representation of topics [97]. In this approach, documents and words are embedded in the same vector space, and the resulting topic vectors are represented in this space. The distance between all these vectors thus indicates their semantic similarity. To set up the Top2Vec function, we used the built-in “deep-learn” option, which should give the best learning performance, and the pretrained embedding model “distiluse-base-multilingual-cased”, which performs well on large datasets in English.

## Results

In this section, using our article set and the referenced WEF Topic Level I and II, we will address the following two key questions: (**RQ1**) which economic topics were prevalent between January and August 2020, and which were *not* and *why*, and (**RQ2**) how did these topics and their associated subtopics evolve and interact *over time* and *around the world* in parallel with the COVID-19 cases?

### Time Pattern of the COVID-19 Topics

**Table 2:** Number of articles for each topic category on the WEF Topic Level I, displayed in reverse order

Topic name	Number of articles	Ratio of articles
Public Policy, Governance and COVID-19	82,967	29%
Developing and Distributing Vaccines	71,293	25%
COVID-19’s Economic Impact	64,041	22%
COVID-19’s Impact on Travel and Trade	37,670	13%
The Virus and the Disease	13,502	5%
The Media’s Role During COVID-19	13,347	5%
COVID-19 Treatment	11,925	4%
New Variants	8,354	3%
COVID-19, Seasonality and Weather	547	0.2%

**Fig. 6:**
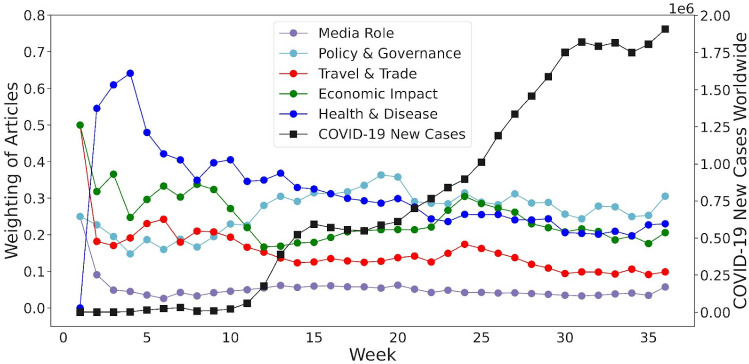
The weighting of articles at WEF Topic Level I relative to the week shows that virus-related topics dominated in the first days of COVID-19, pushing the other relevant topics into the background. Although topics such as *Health & Disease*, *Economic Impact* and *Policy & Governance* were the focus of media articles, none of these topic curves show a consistent upward trend compared to the new cases of COVID-19 worldwide

To answer **RQ1** and the temporal component of **RQ2**, we referred to the WEF Topic Level I that contains nine different COVID-19 related topics, each enriched with a concise description. We calculated the cosine similarity between the embedding vector of news articles and the embedding vector of topic descriptions to determine the proximity of an article to a particular WEF topic. Although there is no uniform threshold for similarity depending on the vector space, we assumed that an article can cover several different aspects and be assigned to different topics as long as the corresponding cosine similarity is at least 0.7. On the one hand, this similarity threshold is comparable to the previous study on a general similarity threshold based on a word embedding dimensionality between 100 and 400 [98], and on the other hand, it is quite strict in our case, as we found that the minimum, mean and maximum similarity values are 0.086, 0.618 and 0.924, respectively. Our sampling of 50 articles each from the *Economic Impact* and *Policy & Governance* topics also confirmed the relevance of the articles to the selected topics. The threshold affects the number of articles classified for each topic listed in Table 2. Nonetheless, as expected, more articles fall into the topics of economics, policy and health. As the health-related topics are similar, we have combined the five topics *COVID-19 Treatment*, *New Variants*, *COVID-19, Seasonality and Weather*, *Developing and Distributing Vaccines* and *The Virus and the Disease* into one topic called *Health & Disease*.

At the beginning of COVID-19, a lot of attention was paid to the virus itself, while other relevant issues such as the economic consequences of the virus received less attention. This observation can be deduced from the articles in the media. Fig. 6 shows the weighting of articles on a given topic in relation to time, i.e., week. New cases of COVID-19 at the global level are plotted on a weekly basis in the graph as a reference. The weighting of a topic is defined as the ratio of the number of articles belonging to that topic to the total number of articles collected in the same period. Note that in the first three weeks, the number of articles collected in the dataset was much less than 500 – more precisely, only 4, 22 and 41 respectively – which could have led to the very large variations in the weighting of the topics. Therefore, no reliable conclusions can be drawn from the first three weeks of data. Overall, *Health & Disease*, *Economic Impact* and *Policy & Governance* are the top three topics throughout the period. At the beginning of 2020, health concerns seemed of primary importance in the public media due to the COVID-19 outbreak. At that time, the enormous economic and social impact of COVID-19 was not yet foreseeable, but only became apparent later. We observe that by week 12, mid-March 2020, the media tended to be “attracted” to new cases of COVID-19 (even though relatively few new cases have been reported), inadvertently ignoring the potential economic impact of the virus. For example, on 13 February 2020, in the middle of week 7, there was a small global peak of 15,153 new cases of COVID-19 according to Our World in Data. This could explain the relatively low media focus on *Economic Impact* and the slightly higher emphasis on *Policy & Governance* at the time, as shown in Fig. 6. Interestingly, the curves for *Health & Disease* and for *Economic Impact* move in almost opposite directions, implying that the economic impact of COVID-19 may only be on the agenda when media attention to new cases of COVID-19 is comparatively low. Meanwhile, the *Policy & Governance* curve, which represents the response to the pandemic over this period, initially rises steadily and has remained relatively flat since then.

As COVID-19 began to spread globally, people and organizations faced many uncertainties and struggled to take the right countermeasures, even at a strategic level. This argument can be drawn by explaining the fluctuations and peaks in the topic curves, as shown in Fig. 6. We assume that these peaks may indicate important events or developments in 2020. However, attributing language use in a text corpus to extra-linguistic events is an interpretive process fraught with uncertainty [55]. Therefore, it is necessary to support interpretation by sifting through samples of the original data. For example, in Fig. 6, the curve for *Economic Impact* has a peak in week 8. The peak implies that in week 8 – that is in late February – the debate about if and how the COVID-19 crisis has consequences for the global economy intensified. An example would be an article on CNBC about the airline Air France-KLM, the first sentence of which reads as follows: “Air France-KLM warned on Thursday of a 150 million to 200 million euro ($162 million to $216 million) hit to earnings by April as it contends with the China coronavirus epidemic’s ‘brutal’ impact on the airline industry”^6^. A closer look at a sample of 50 articles marked *Economic Impact* in week 8 shows that a large proportion of these articles refer to corporate earnings reportings or press releases of profit and loss statements for 2019 or for the fourth quarter of that year. It is worth noting that companies outside the aviation industry have also recognized COVID-19 as a potential economic issue, but mostly considered it as an “unknown factor”. A short article on MarketWatch quotes a U.S. official saying that “it is too soon to say how the [COVID] outbreak might impact the U.S. economy”^7^. It is thus striking that at the end of February 2020, the COVID-19 crisis was still seen as an economic problem that only affected China. At that time, COVID-19 was not yet considered a global economic crisis.

Later in week 19, we examined a sample of 50 articles, this time on the topic of *Policy & Governance*. As the WEF authors note in their description of the topic *Public Policy, Governance and COVID-19*, governments must decide which “combination of restrictions, tailored to circumstances, is needed to prevent transmission”^8^. This is reflected in the sample, for example in two articles from express.co.uk and CNBC, respectively, referring to the U.K. government’s decision to ease up the lockdown. The later one, published on CNBC on 10 May 2020, states that “The U.K. government has taken its first tentative steps to ease some strict coronavirus lockdown measures and slowly begin to reopen society and the economy”^9^. In addition, companies and individuals face extreme economic uncertainties associated with the pandemic, therefore “managing response measures and mitigating impacts” must be at the heart of “effective public policy and governance”^10^. Overall, quarterly reports – such as the one from which the above quote is taken – and articles belonging to closely related text types make up about 50% of the sample. The peak in the topic *Policy & Governance* in week 19 is therefore at least partly due to the publication of a large portion of quarterly reports covering the first three months of 2020. This temporal peak should therefore not be over-interpreted. Despite this caveat, the quarterly reports in the sample reflect how companies have observed and responded to government policies – some explicitly as in the example quoted above, others more implicitly.

**Fig. 7:**
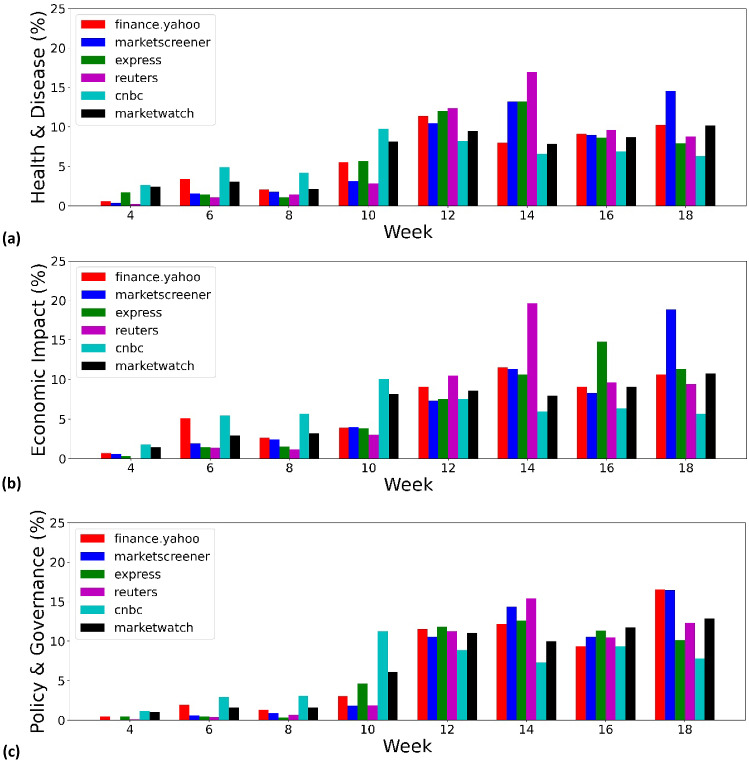
The herd behavior of the media can be observed by examining how they distribute their “publishing efforts” depending on external events. The proportion of articles from the media is presented per week on the topics of (**a**) *Health & Disease*, (**b**) *Economic Impact* and (**c**) *Policy & Governance*

### Herd Behavior of the News Media

The uncertain situation caused by COVID-19 is certainly due to the unprecedented scale of the pandemic and is also “amplified” by the herd behavior of the media, i.e. specialized business and economic news media tended to report on the same topics during the same period, which prevents a comprehensive view of the crisis. To examine the attention paid by different media outlets to coverage of the three main topics mentioned above, i.e., *Health & Disease*, *Economic Impact* and *Policy & Governance*, Fig. 7 shows the proportion of articles published weekly on a media platform on a given topic in relation to the total number of articles published on the same platform on that topic in the first days of COVID-19, i.e., from the end of January to the beginning of May 2020. This gives us an indirect measure of how much “effort” the media have put into reporting on the development of the pandemic. Referring to Fig. 2, we have selected the six media platforms that contribute the most to our dataset: Yahoo Finance (financial news), MarketScreener (financial analyst), express.co.uk (tabloid), Reuters (newswire), CNBC (quality news) and MarketWatch (financial news). These different sources of information provide a comprehensive overview of news coverage in terms of news types and quality. Interestingly, these media platforms show a herd behavior, i.e., the same tendency in the relative number of publications, regardless of the chosen topic. Combined with Fig. 6, it shows that the only trigger for the media to report on these topics is the new cases of COVID-19 worldwide. We have not plotted Fig. 7 with data up to the end of August 2020 because of the heterogeneity of the number of articles collected by Yahoo Finance, which has increased from 2,805 in week 25 to 10,787 in week 30. In fact, the herd behavior of the other five media platforms is clearly visible until the end of August 2020. We believe that the herd behavior of the news media can lead them to focus only on the most popular topics at a given time, neglecting to report and reflect on issues that are not relevant in the short term but are important in the long term [82]. This argument is further substantiated using the WEF Topic Level II, where we see the topics that do not surface following the outbreak of COVID-19.

**Table 3:** Number of articles for each topic category on the WEF Topic Level II, displayed in reverse order

Topic name (short name)	Number of articles	Ratio of articles
Financial and Monetary Systems (FMS)	232,703	80%
Future of Media, Entertainment and Sport (FMES)	196,144	68%
Banking and Capital Markets (BCM)	152,812	53%
Retail, Consumer Goods and Lifestyle (RCGL)	148,318	51%
Vaccination (VAC)	148,191	51%
Pandemic Preparedness and Response (PPR)	143,552	50%
Digital Economy and New Value Creation (DENVC)	134,745	46%
Public Finance and Social Protection (PFSP)	119,739	41%
Workforce and Employment (WE)	114,190	39%
Advanced Manufacturing and Production (AMP)	107,364	37%
Aviation, Travel and Tourism (ATT)	103,638	36%
The Digital Transformation of Business (DTB)	102,451	35%
Ageing (AGE)	98,263	34%
International Trade and Investment (ITI)	94,308	33%
Innovation (INN)	91,284	31%
Global Health (GH)	73,699	25%
Science (SCI)	67,221	23%
Future of Health and Healthcare (FHH)	45,560	16%

For a finer examination of the topics (**RQ1**), we continued the analysis of the articles on the WEF Topic Level II. We selected 18 WEF topics that are highly relevant to our dataset, as they overlap with the main topics we identified based on the most important words in the articles. Note that the descriptions of these WEF topics may differ from those of the WEF Topic Level I, even when the topic names are similar. For example, the topic *Vaccination* on the WEF Topic Level II is explained by several key issues, namely *Trust, Misinformation and Health*, *Vaccine Hesitancy*, *Economic and Business Implications of Vaccination*, *Vaccinating for Security*, *Vaccinating Against Pandemics* and *Vaccination and the Workplace*. For each key issue, the WEF provides a brief description explaining the scope of the issue, which affects the text embedding and classification of the articles. Since the minimum, average and maximum similarity values for articles and topics are 0.186, 0.686 and 0.922 respectively, we claim that each article can contain multiple topics as long as they meet the similarity threshold of 0.7, as in the previous argument. The number of articles classified for each topic is shown in Table 3. The economic topics *Financial and Monetary Systems* and *Banking and Capital Markets* rank first and third, respectively. The health-related topics *Vaccination* and *Pandemic Preparedness and Response* rank fifth and sixth, respectively. Each of these topics accounts for about half of the articles in the dataset. There is no specific *Policy & Governance* topic on the WEF Topic Level II.

**Fig. 8:**
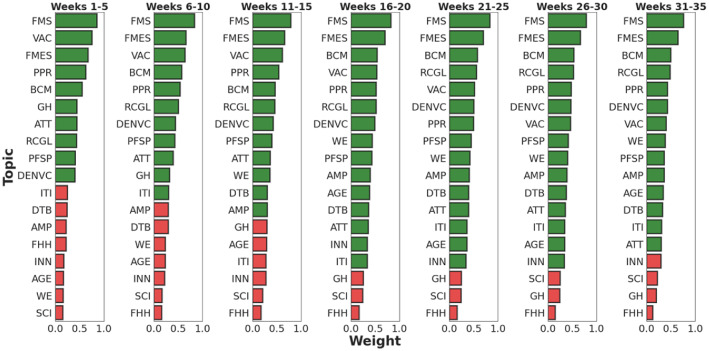
The herd behavior of the media prevents the potentially important topics from being discussed in the early stages of COVID-19. Ranking of the percentage of articles on specific topics is shown in a five-week window starting in 2020 and lasting 35 weeks. From left to right, the range of topics that have not surfaced has narrowed over time. Green: topics with an article ratio of more than 0.3; Red: topics with an article ratio of 0.3 or less

Our analysis on the WEF Topic Level II underpins the observation of herd behavior of the media, as some potentially important topics are not widely discussed in the early phase of COVID-19. To get a clear picture of which topic has not surfaced, Fig. 8 assesses the percentage of articles on a topic in a five-week window starting in 2020 and lasting 35 weeks. The threshold for determining the topic to surface is set at 0.3. As can be seen, up to week 15, the top three topics were always FMS, VAC and FMES. The frequency of mention of the topic VAC reflects the media’s interest in immediate protection measures for COVID-19. From week 16 onwards, this topic was displaced by BCM, demonstrating the gradual increase in media interest in the economic impact of COVID-19. Interestingly, the number of topics that do not surface, marked in red in Fig. 8, decreases over time. For example, in the first 10 weeks, the topics of DTB and WE did not receive much attention. Although they are directly related to the impact of COVID-19 on our digital economy and labor markets, they were not covered extensively in the media during these first days. INN, SCI, GH and FHH remained relatively undiscussed throughout the eight-month period. The reason for the “absence” of the first two could be the very broad range of the topics, which include “innovative solutions that address the most pressing issues, from pandemics to climate change”^11^. Nevertheless, some of their issues do not need to be addressed as urgently compared to COVID-19. A pandemic of this magnitude is also a major challenge for GH and FHH, as the current capital-intensive, hospital-centric model of health care is not conducive to promoting the well-being of all people. Fundamental change is needed in existing health systems with “a coordinated and data-enabled approach” to “connect with patients and communities”^12^. Clearly, for this to happen, it will take some time for the public media to take into account the lessons learned from COVID-19.

**Fig. 9:**
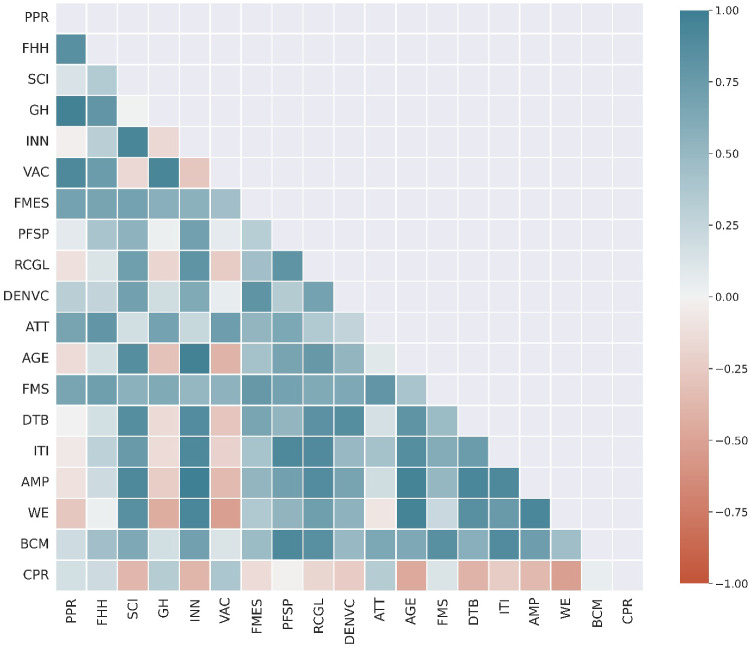
Topic correlations show the “dependence” of relevant topics in media news for the same period from January to August 2020. The correlations between these topics and the COVID-19 positivity rate (CPR) are also shown for reference. 1: completely positive correlation; -1: completely negative correlation

**Fig. 10:**
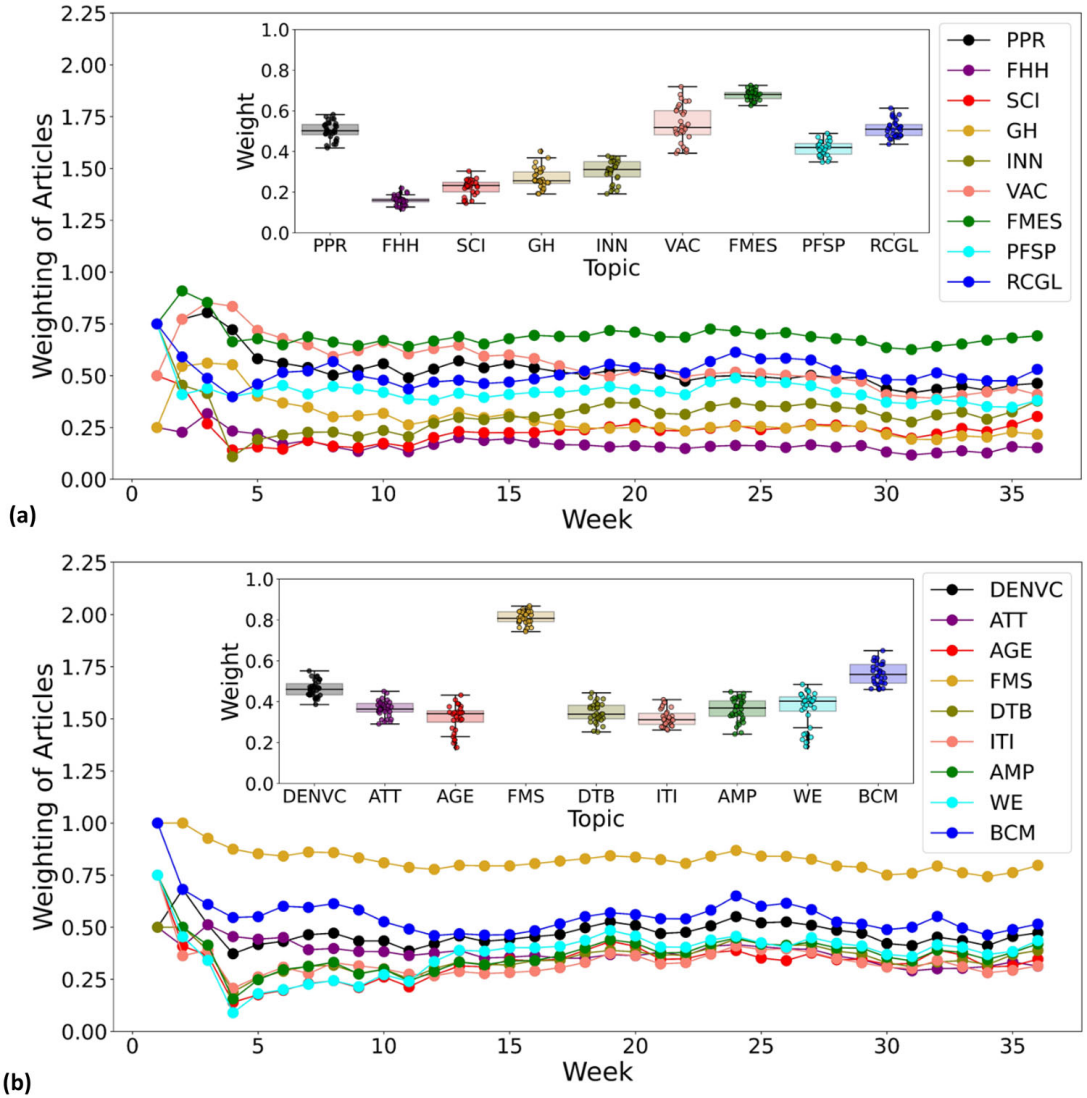
The development of COVID-19-relevant topics shows the “interdependence” over time. The weighting of the articles on the 18 topics selected from WEF Topic Level II is shown as a function of the week: (**a**) topics mainly related to health and social life, and (**b**) topics related to the economy and the financial system. The inset shows the corresponding variation of article weighting for each topic from week 4 to week 36

### Topic Interaction over Time

Despite the herd behavior of the media, we have seen from the media coverage in the first eight months of COVID-19 the spread of the impact of the pandemic on all aspects of our economy and society. These potential areas of influence are interlinked over time (**RQ2**). Based on the proportion of articles on a given topic per week, Fig. 9 plots the pairwise correlations for the 18 topics listed in Table 3. Note that the correlation does not measure the similarity in the absolute number of articles reported, but the relevance of the change in the number of articles reported on different topics. Correlations between these topics and the COVID-19 positive rate (CPR)^13^ are also calculated. The first observation is that CPR has a relatively higher positive correlation only for the health-related topics such as PPR (0.17), FHH (0.2), GH (0.32), VAC (0.38) and ATT (0.33). This is not surprising as the media tends to follow the immediate impact of the outbreak. In addition, CPR shows only low positive correlations with FMS (0.12) and BCM (0.04), but negative correlations with other topics. Note that many of the topics are “intertwined”, meaning that they are often covered simultaneously in the media. For example, the two economic topics BCM and FMS have a strong positive correlation, i.e., 0.84. At the same time, they seem to complement coverage of several other topics, such as ITI (BCM: 0.89; FMS: 0.61), PFSP (BCM: 0.91; FMS: 0.69) and RCGL (BCM: 0.85; FMS: 0.62). The health-related topics PPR and VAC have a strong positive correlation of their own, i.e., 0.91. However, they are weakly correlated with BCM (PPR: 0.19; VAC: 0.12), but significantly more strongly correlated with FMS (PPR: 0.66; VAC: 0.55). This could be due to the closer link between FMS and the economic risk of a global pandemic. Interestingly, WE is significantly and positively associated with the two topics SCI and INN, suggesting the importance of technological factors in strengthening labor market dynamics. Moreover, WE is negatively correlated with health-related topics such as PPR ($-0.27$), GH ($-0.44$) and VAC ($-0.52$). The fact that WE was not reported as equally important as health-related topics at the same time may indicate that governments and relevant authorities had not taken sufficient notice of, or were not yet fully prepared for, the potential impact of COVID-19 on the labor market. They may not have the energy to address the response in these areas until the epidemic is relatively “stable”. Similar observations can be made for the following topics: RCGL (PPR: $-0.11$; GH: $-0.18$; VAC: $-0.24$), DTB (PPR: $-0.0075$; GH: $-0.15$; VAC: $-0.28$), ITI (PPR: $-0.06$; GH: $-0.15$: VAC: $-0.2$) and AMP (PPR: $-0.11$; GH: $-0.23$; VAC: $-0.35$). They also suggest that our society as a whole was not fully prepared for a pandemic of this magnitude, at least in the early 2020s.

We further assume that the “interdependence” between COVID-19-relevant topics shown in Fig. 9 can be viewed by plotting the evolution of the various health and economic topics over time. To visualize the temporal patterns of the WEF Topic Level II (**RQ2**), Fig. 10 plots the article weights for specific topics against weeks. Most topics related to health and social life are shown in Fig. 10a, while most topics related to the economy and the financial system are shown in Fig. 10b. The inset shows the calculated median weights of the articles and their variations between weeks 4 and 36. As shown in Fig. 10a, different aspects of health and social life receive different levels of attention. FMES remains at a consistently high level, partly due to the widespread public interest in the topic and partly probably due to the very broad scope of the topic itself. Somewhat surprisingly, PPR and VAC show a similar downward trend over time. This seems to contradict the trend for coronavirus and vaccine development, but one must also consider the total amount of media coverage of other related topics and the relative weight of individual topics. PFSP and RCGL moved at a similar pace during this period, receiving the most attention in week 24. The proportion of other topics in Fig. 10a is essentially less than 0.4. Turning to Fig. 10b, it is very interesting, but expected, that topics related to the economy and the financial system show a high trend of correlation and comparability. Media interest in these topics gradually increased as the epidemic “stabilized” and remained at a relatively flat level after an initial period up to week 12. Here, the great weight of FMS is probably due to its extensive topic coverage. The topic BCM had a more pronounced peak in weeks 8 and 24 than in Fig. 6. This may have had a strong influence on the coverage of the topic DENVC, which is driven by COVID-19 and is increasingly attracting the attention of our society. The share of other relatively specific economic topics in Fig. 10b is less than 0.4. In general, the dominant topics, namely PPR, VAC, FMES, PFSP, RCGL, DENVC, FMS and BCM, do not seem to leave much room for other topics in the news media.

**Fig. 11:**
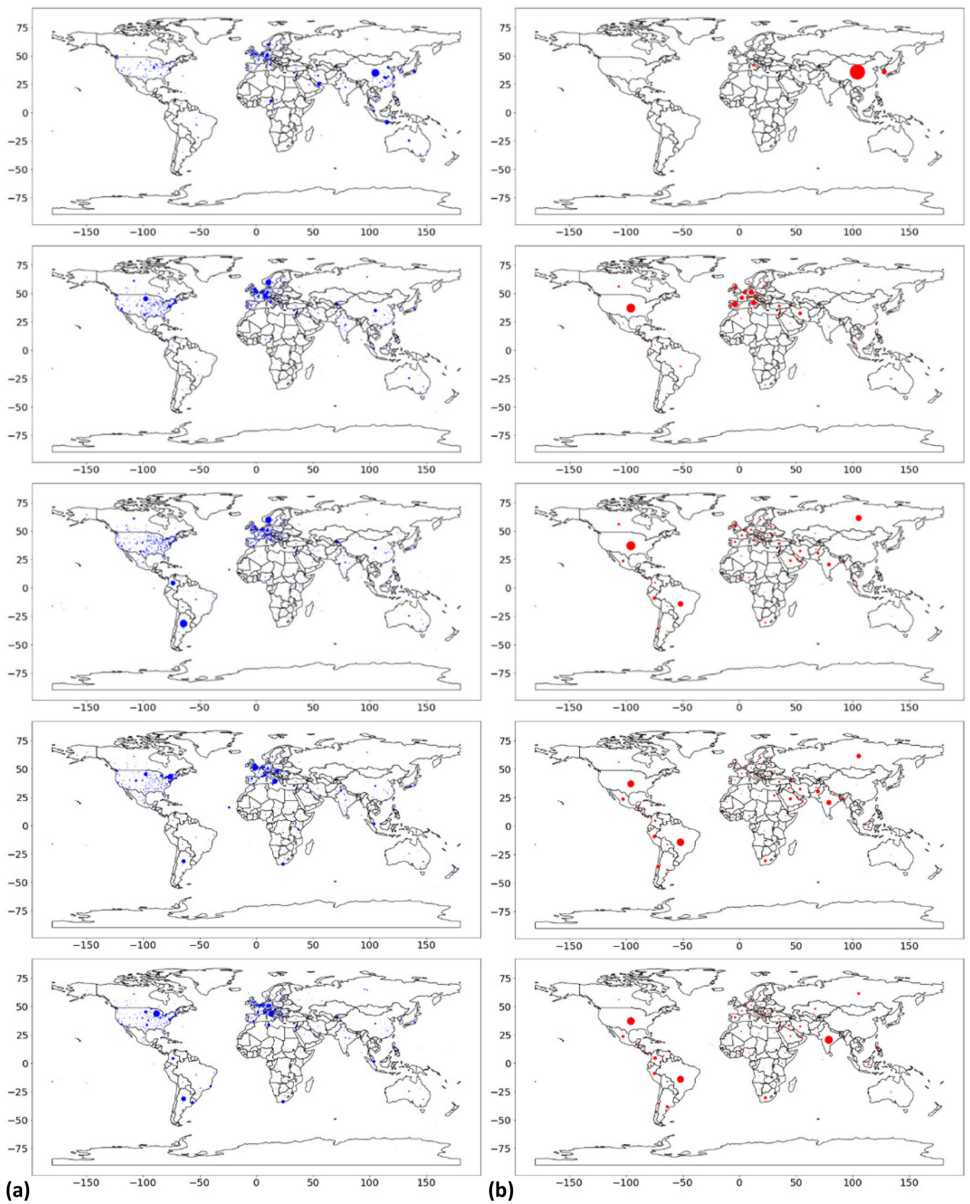
The development of COVID-19-relevant topics shows the “interdependence” across space. Global dispersion of (**a**) the topic DENVC reported in the news media and (**b**) the COVID-19 new cases according to Our World in Data. From top to bottom, the timing corresponds to the 8*th*, 13*th*, 19*th*, 24*th* and 32*nd* weeks of 2020, respectively. The dots in the graph, whose size represents (**a**) the frequency of mention of the topic DENVC or (**b**) the number of new cases of COVID-19, have been resized on the same scale to allow comparison of the intensity of the hotspots. In (**b**), geographical coordinates are shown only at the country level

**Fig. 12:**
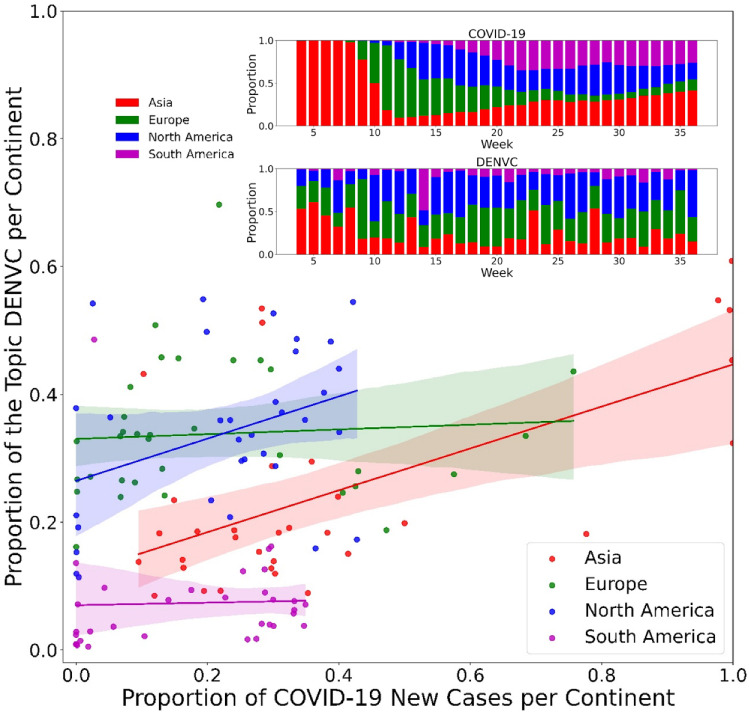
The correlation that the continent shows between the proportion of the topic DENVC in the media and the proportion of COVID-19 new cases supports the temporal and spatial “dependence” of the two. Each point represents a value for the week between week 4 and week 36. The linear regression line shows the trend of the correlation. The inset shows the proportion of COVID-19 new cases or the topic DENVC per continent in relation to the week. Only Asia, Europe, North and South America are shown, as they are the focus of the media

### Topic Interaction over Space

The spread of COVID-19-relevant topics around the world also closely follows the steps of the virus, showing a time lag in media coverage at the global level. To visualize the spatial patterns of the WEF Topic Level II (**RQ2**), we selected the topic DENVC from Fig. 10 to show its hotspots at the geographical level. This topic was selected as an example because it represents an important aspect of the changing economic environment in 2020, namely the global digital revolution accelerated by COVID-19. This is also shown by the fact that the weight of the topic fluctuates around 0.5, meaning that the number of articles assigned to this topic group is sufficiently large (*n* = 134,745). We examined articles in the weeks corresponding to the peaks shown in Fig. 10b, i.e., weeks 8, 13, 19, 24, and 32. Fig. 11a shows the identified hotspots on the world map. For reference, Fig. 11b shows the new cases of COVID-19 reported globally in the same week. Interestingly, it can be observed that the global distribution of the topic DENVC reported in the media largely matches the distribution pattern of new COVID-19 cases. In early 2020, the first coronavirus cases occurred in China. As the virus spread worldwide, media coverage of the topic DENVC also traveled further West, to Europe and the U.S., eventually reaching South America. Therefore, it is reasonable to assume that the topic of media coverage is closely related to the coronavirus and its potential impact on the economic model. However, from week 19 to week 32, we also note an intensification of the COVID-19 epidemic in countries such as Russia and India, but cannot see a similar pattern in the geographical distribution of the topic DENVC. This could be due to the consistent focus of the English-language media on the U.S. and Europe. A similar pattern of dissemination can be observed for other topics such as RCGL. It seems that the pandemic “predicts” topics in media coverage: If a topic is of concern in China when cases rise there at $t_0$, the same topic is likely to be on the agenda when cases rise in the U.S. and Europe at $t_1$ and in South America at $t_2$. In this context, we believe that close monitoring of global news coverage of viruses and related topics can help companies and policymakers prepare for the issues posed by an ongoing pandemic. Thus, monitoring the news landscape on a global scale can support the prevention of social and economic crises such as COVID-19.

To support these arguments based on Fig. 11, Fig. 12 shows the relationship between the proportion of the topic DENVC in the media and the proportion of COVID-19 new cases viewed by continent. Only values between week 4 and week 36 are used, and a regression line is plotted to show the corresponding trend. Although the data exhibit a degree of dispersion, positive correlations across most continents show that new cases of COVID-19 acted as a “driving” force for media coverage of the topic DENVC. This phenomenon is particularly evident in Asia and North America. As further shown in the inset of Fig. 12, the proportion of the topic DENVC in the global news media alternates with the proportion of new cases of COVID-19 across the continents of Asia, Europe, North America and South America. A similar rhythm can be observed in Asia and North America in particular. This indicates that during COVID-19, the media at least tried to monitor the social dynamics and market changes caused by the virus and remained alert to the development of new economic models. Therefore, environmental scans as well as forecasts need to include global media coverage to ensure appropriate development and implementation of business and public policies. To help further understand the hotspots in Fig. 11a, we used the topic modeling approach Top2Vec, which aims to detect micro-topics described by news articles that are part of the topic DENVC. Using week 19 as an example, a total of 5,795 articles were found under the topic DENVC. For each identified micro-topic, a list of semantically meaningful keywords and representative articles is generated. Based on this, we identified a large number of key topics of interest to the media. Some are more related to health and digital healthcare, e.g., mask wearing, coronavirus testing and vaccination, pharmaceuticals and their response to coronavirus, and contact tracing for coronavirus. Some are more relevant to the economic consequences of COVID-19, e.g., lockdown and reopening, the impact of COVID-19 on the company’s financial returns, or stock markets and investments. There are also some more general micro-topics, such as accusations and sanctions against China, virtual meetings, or online learning. Most of these micro-topics link well to the topic DENVC, showing the broad impact of COVID-19 on all aspects of the digital economy. Therefore, closely monitoring the development of these micro-topics at the global level can facilitate rapid adaptation to social dynamics and market changes from an economic and political perspective.

## Discussion and Contribution

The analyses conducted in this study involve the ongoing pandemic, thus the corresponding results are closely related to real events, namely COVID-19 cases. This is shown by the extensive media coverage of the pandemic in China at an early stage and the growing concern about various aspects of socio-economic life under the pandemic around the world. News coverage of the pandemic follows the rise and fall of COVID-19 positive cases both in time and space. The data show that the pandemic “predicts” the salience of topics in news coverage across the globe. This observed trend holds significant implications for policymakers and businesses that need to prepare for the upcoming challenges of a crisis [99]: monitoring global media coverage of the same topic, even across languages, provides decision-makers in organizations with a glance into the future, i.e., which topics will become relevant to their constituents in the short term. This environmental scanning via automated analyses of global news coverage gives them the often-lacking time advantage to address new or unexpected challenges that arise from crises.

**Fig. 13:**
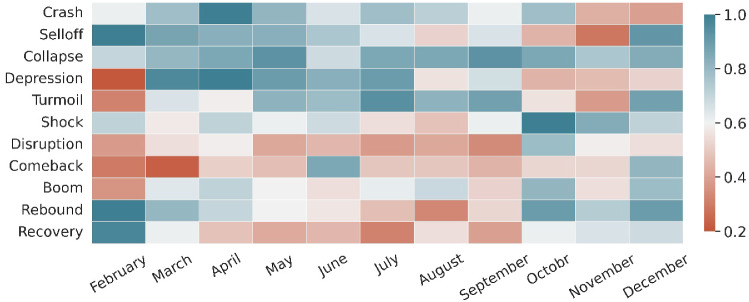
The news media reported more positively on economic topics with the development of COVID-19. In 2020, the terms most closely associated with the concept of *Recession* have changed over time from more negative to more positive. The color bar shows the relevance of these terms to *Recession*

Our analyses demonstrate that in the first quarter of 2020, when COVID-19 first broke out, few media and publishing institutions were able to understand the potentially devastating impact of the epidemic, as evidenced by the low variety of topics. Despite the 24/7 news cycle and the technically almost limitless space of the Internet, the media arena is limited in its carrying capacity [81]. Overly represented topics in the news such as health and disease overshadowed related subtopics. Since news media tend to report homogeneously and thus imitate each other’s topics, the media coverage of the crisis was relatively uniform, potentially ignoring issues that may only be relevant to a minority today but could develop into significant issues in the long run [82]. As media act as agenda-setters in society [80], their herd behavior also prevented citizens from receiving independent information to prepare for a pandemic and its enormous economic and social impact. However, we also found that the further a crisis matures, i.e., the less novel it is, the more diverse the topics in the news become. The uniformity of media coverage of the pandemic is thus likely coupled with the temporal maturation of the crisis in the life cycle [4]. This is illustrated by the fact that the topic *Workforce and Employment* only gained salience at a later stage of the first wave of the pandemic. However, the negative correlations with health-related topics also suggest that the news media tended to discuss this topic in isolation from the health consequences of the pandemic, which seems paradoxical given today’s knowledge. Thus, monitoring global issue discussions in combination with their related topics and silent issues gives decision-makers a proper basis for decision-making in uncertain times. News media can avoid herd behavior by prioritizing other news values such as good news or magnitude [82] to report on a more diverse set of topics and thus offer unique information to potential new or minority audiences. This would also meet the need of citizens to seek more, and more diverse, information in crisis situations to make sense of the events [3]. Ultimately, a more pluralist news landscape promotes diverse democratic discourse in society rather than news fatigue [5], which is particularly important in a pandemic where policymakers limit fundamental rights.

So far, we have looked at the COVID-19 discussion on a factual level and seen how it developed in terms of relevant topics and locations. However, we have not touched a more subjective dimension of the data, namely the tone of news media coverage. An interesting research question here would be: How positively (negatively) did the news media report on COVID-19 related topics? To get to the bottom of this question, we briefly examined an example, namely the economic recession caused by COVID-19. We trained a word embedding model [100–102] on the entire dataset and selected the 10 closest neighbor terms of *Recession* based on distributional similarity. We then trained a word embedding model for each month and compared how the similarity between *Recession* and each of the neighboring terms changes over time. Monitoring economic terms and their evolution over time is an important knowledge base for policy and financial decision-makers, as it could influence the policy agenda and the volatility of financial markets [103]. Fig. 13 shows the results obtained. Some of these terms, such as *Crash*, *Selloff* and *Depression*, are clearly negative. Others, such as *Comeback*, *Boom* and *Rebound*, are more positive. On the one hand, it can be seen that the negative terms are more strongly associated with *Recession* in the first months of the pandemic. On the other hand, the positive terms tend to predominate at the end of 2020. These analyses show that while the severity of the pandemic increased from wave to wave, the tone in the media regarding economic development did not follow the same pattern. Instead, the public media reflected the complexity of the large network of topics intertwined with COVID-19 that has evolved over time and space, which also helped to shape the overall tone in the media.

The significance and contribution of this study to data science lies mainly in the novel application of a set of advanced NLP techniques to retrieve key information from long news article texts. The proposal of this analysis framework goes beyond existing research that either trains only an embedding model based on news [104] or directly uses the existing models from Spark NLP to analyze them [105]. Instead of the traditional Word2Vec embedding and LDA-based topic modeling methods [106], our analytical approach is based on the state-of-the-art sentence embedding techniques Sentence-BERT and the density-based clustering algorithm Top2Vec, which attempt to encode all textual information into different economic topics as defined by the WEF. This is a step up from a more recently published study [107], which used similar NLP techniques but only encoded the title and summary of online news articles. The power of NLP models based on transformers ensures that contextual information is correctly embedded in a mathematical space. The use of newer, pre-trained models can also help to increase the accuracy of text classification. However, since the length of the input to the sentence embedding model is limited, we need to split the article into several parts, encode them separately and average the vectors obtained to get the final text embedding. This could result in the essential information contained in the text being lost during the averaging process. We improved the retention of key information by considering article titles and embedding TF-IDF-identified keywords. The next step could be to embed summaries to further improve article classification. Overall, the established text analysis framework is generally workable and provides a new approach for extracting topics in long media articles.

## Conclusions and Outlook

In this work, we have investigated the economic aspects of COVID-19 in the first wave by exploring a large dataset of news articles using state-of-the-art NLP techniques. We made innovative use of advanced sentence embedding methods and COVID-19 topics defined by WEF’s Strategy Intelligence. We have shown which topics dominated between January and August 2020, which topics gradually gained attention during this period, and which topics were marginalized by the media’s focus on more immediate issues. The interaction between these topics and their evolution over time and space were used as agenda-setters for public discourse. Our analyses showed that in the early days of COVID-19, health-related topics were the focus of media coverage, while other topics, such as the economic impact of the virus, tended to take a back seat. However, as COVID-19 became established as a pandemic, the media were gradually prompted to cover all social and economic aspects, and the spread of news topics largely coincided with the spread of the virus. In this context, our research points to the importance of monitoring the global news landscape to help businesses and society prepare to prevent the social and economic consequences of COVID-19. This is important to effectively respond to future social dynamics and market changes caused by health crises like this one. The herd behavior of the news media observed in this pandemic is a reminder of the need for the media to establish an independent agenda.

In a future study, we are interested in developing an analytical framework for organizational management learning under the COVID-19 pandemic. As the report for the first quarter of 2020 included in the dataset shows, some companies performed strongly when the coronavirus hit, while others faced a severe crisis. Organizational management can therefore learn extensively from the ongoing pandemic, which is important to improve their resilience and ability to adapt quickly [108]. Studying the behavior of these companies will help in taking timely and effective management actions in case of similar crises in the future. In this context, we extend our dataset collecting relevant news articles to date and propose to link the behavior of companies under COVID-19 in media articles with their financial performance (e.g., survival and growth in sales, severity of loss and time of recovery of the stock prices) through NLP. This involves using NER and fuzzy recognition techniques to identify companies, combining topic modeling models for aspect extraction, and sentiment analysis of our own collected data from Twitter to quantify the behavior of companies and consider their impact on market reactions. Connections between companies can be established through community detection and network analysis methods. The knowledge graph thus created will help us better understand corporate crisis response initiatives and the reasons for their success and failure.

## Footnotes

1 COVID-19 Public Media Dataset by Anacode (https://www.kaggle.com/datasets/jannalipenkova/covid19-public-media-dataset-anacode).

2 COVID-19 new cases by Our World in Data (https://github.com/owid/covid-19-data/tree/master/public/data).

3 COVID-19 Open Research Dataset Challenge (https://www.kaggle.com/allen-institute-for-ai/CORD-19-research-challenge).

4 World Economic Forum’s Strategic Intelligence (https://intelligence.weforum.org/topics).

5 https://huggingface.co/sentence-transformers.

6 https://www.cnbc.com/2020/02/20/air-france-says-coronavirus-impact-could-climb-above-200-million.html.

7 https://www.marketwatch.com/story/feds-clarida-economic-outlook-is-a-good-picture-2020-02-20.

8 https://intelligence.weforum.org/topics/a1G0X000006O6EHUA0?tab=publications.

9 https://www.express.co.uk/news/uk/1278528/pmqs-boris-johnson-keir-starmer-coronavirus-lockdown-end-UK-latest-covid-19 and https://www.cnbc.com/2020/05/10/coronavirus-uk-boris-johnson-outlines-plan-to-reopen-economy.html.

10 https://intelligence.weforum.org/topics/a1G0X000006O6EHUA0?tab=publications.

11 https://intelligence.weforum.org/topics/a1G0X000006DO7RUAW?tab=publications.

12 https://intelligence.weforum.org/topics/a1Gb00000038u3nEAA?tab=publications.

13 COVID-19 new cases and new tests by Our World in Data (https://github.com/owid/covid-19-data/tree/master/public/data).
